# Systematic Administration of B Vitamins Alleviates Diabetic Pain and Inhibits Associated Expression of P2X3 and TRPV1 in Dorsal Root Ganglion Neurons and Proinflammatory Cytokines in Spinal Cord in Rats

**DOI:** 10.1155/2020/3740162

**Published:** 2020-02-10

**Authors:** Duan-Duan He, Yu Gao, Shan Wang, Zhong Xie, Xue-Jun Song

**Affiliations:** ^1^Department of Anesthesiology, Peking University Third Hospital, Beijing, China; ^2^Department of Anesthesiology, Peking University International Hospital, Beijing, China; ^3^SUSTech Center for Pain Medicine and Medical School, Southern University of Science and Technology, Shenzhen, Guangdong, China

## Abstract

**Background:**

Treatment of diabetic neuropathic pain (DNP) continues to be a major challenge, and underlying mechanisms of DNP remain elusive. We investigated treatment effects of B vitamins on DPN- and DNP-associated alterations of neurochemical signaling in the nociceptive dorsal root ganglion (DRG) neurons and the spinal cord in rats.

**Methods:**

DNP was produced in male, adult, Sprague Dawley rats by single i.p. streptozotocin (STZ). Western blot analysis and immunohistochemistry were used to analyze protein expressions in DRG and ELISA to measure the proinflammatory cytokines in the spinal cord. Behaviorally expressed DNP was determined by measuring the sensitivity of hindpaw skin to mechanical and thermal stimulation.

**Results:**

There were 87.5% (77/88) rats which developed high blood glucose within 1-2 weeks following STZ injection. Of which, 70.13% (*n* = 54/77) animals exhibited DNP manifested as mechanical allodynia and/or thermal hyperalgesia. Intraperitoneal administration of vitamins B1/B6/B12 (100/100/2 mg/kg, one or multiple doses) significantly attenuated DNP without affecting the blood glucose. Expressions of P2X3 and TRPV1 in CGRP-positive and IB4-positive DRG neurons as well as the interleukin-1*β*, tumor necrosis factor-*α*, and nerve growth factor in the lumbar spinal cord were greatly increased in DNP rats. Such DNP-associated neurochemical alterations were also greatly suppressed by the B-vitamin treatment.

**Conclusions:**

B-vitamin treatment can greatly suppress chronic DNP and DNP-associated increased activities of P2X3 and TRPV1 in DRG and the spinal proinflammatory cytokines, which may contribute to the pathogenesis of DNP. Systematic administration of B vitamins can be a strategy for DNP management in clinic.

## 1. Introduction

Diabetic neuropathic pain (DNP) is one of the most common chronic complications in patients with diabetes mellitus [1, 2]. DNP develops in the later stage of diabetes and affects about 10–26% of patients with diabetes [3–10]. Although hyperglycemia is considered to be a major pathogenic factor in the development of diabetic neuropathy, mechanisms of this severe and intractable complication are not well understood. Studies have implicated multiple processes as possible aetiologies underlie the pathogenesis of DNP, including hyperglycaemia-induced damages to nerve cells and decreased neurovascular flow [11]; loss of myelinated and unmyelinated fibres [12, 13]; elevated production of the proinflammatory cytokines tumor necrosis factor (TNF)-*α*, interleukin (IL)-1*β*, and IL-6 [11, 14–16]; perturbations in growth factors such as nerve growth factor (NGF), insulin-like growth factor, and neurotrophin 3 [11, 16]; immune dysfunction [17]; activation of ephrinB-EphB receptor signaling in the spinal cord [18]; and oxidant stresses in mitochondria and endoplasmic reticulum [11, 16]. Despite these efforts over the past decades, the specific cellular and molecular mechanisms underlying DNP pathogenesis remain elusive, and clinical approaches for DNP treatment are limited.

The B vitamins, thiamine (B1), pyridoxine (B6), and cyanocobalamin (B12), have been reported to be effective in treating certain chronic painful disorders, such as neuropathic pain [19, 20], trigeminal neuralgia [21], diabetic neuropathy [22, 23], and rheumatoid arthritis [24]. It has also been reported that chronic exposure to thiamine, pyridoxine, and cyanocobalamin protects neurons from glutamate or NMDA-mediated excitotoxicity in vitro [25, 26]. We have recently demonstrated that B vitamins can potentiate acute morphine antinociception and attenuate chronic morphine tolerance, possibly by inhibiting glial activation and suppressing proinflammatory cytokine production [27]. In addition, improved absorption and synthesis of B vitamins, as achieved by orally administered galactooligosaccharides (GOS), could suppress microglia activation and regulate several inflammatory-related factors in mice [28]. Diabetic neuropathic pain does not respond well to commonly used analgesics such as opioids or nonsteroidal anti-inflammatory drugs [29–31], posing a major challenge in the clinical management of diabetic pain. Based on existing evidence, we hypothesized that the B vitamins, B1, B6, and B12, might be able to alleviate DNP and thus investigated possible treatment of B vitamins in DNP. In the present study, we tested whether systematic administration of a combination of B vitamins (VBC) including B1, B6, and B12 can alleviate neuropathic pain in a rat model of type I diabetes mellitus induced by streptozotocin (STZ) in rats. Furthermore, we investigated whether VBC can regulate P2X3 and TRPV1, two important membrane proteins associated with DNP, in the dorsal root ganglion (DRG) neurons, as well as whether VBC administration can regulate mediators of inflammatory pain (IL-1*β*, TNF-*α*, and NGF) in the spinal cord. Our results demonstrated that VBC treatment can significantly attenuate thermal hyperalgesia and mechanical allodynia in STZ-induced diabetic rats and suppress the increased expression of P2X3 and TRPV1 in DRG, and the increased level of IL-1*β*, TNF-*α*, and NGF in the lumbar spinal cord, in STZ-induced diabetic rats.

## 2. Methods

### 2.1. Animals

We purchased adult, male, Sprague Dawley rats (200–220 g-wt) from the Department of Laboratory Animal Science of Peking University Health Science Center. The rats were housed in groups of four to five in plastic cages with soft bedding and free access to food and water under a 12 h day/12 h night cycle. Our experimental procedures and animal use were conducted in accordance with the regulation of the ethics committee of the International Association for the Study of Pain and approved by the Animal Care and Use Committee at Peking University Health Science Center. All surgeries were performed under anesthesia with sodium pentobarbital intraperitoneally (50 mg/kg, i.p.).

### 2.2. STZ-Induced Diabetic Neuropathic Pain Model

Diabetes was induced in rats, after an overnight fast, by a single intraperitoneal injection (i.p.) of STZ (Sigma-Aldrich, St. Louis, MO, USA), 70 mg/kg, freshly dissolved in 0.1 mol/L citric acid buffer (CAB, pH 4.5). Animals in the sham group received an injection of an equivalent volume of citrate buffer. A single or multiple measurements of blood glucose were made after injection of STZ in each of the animals. The blood samples were collected from the tail vein blood vessel. Onset of diabetic conditions was defined as glucose levels >16.6 mmol/L.

### 2.3. Evaluation of Mechanical Allodynia and Thermal Hyperalgesia

Mechanical allodynia in DNP rats was determined by measuring the incidence of foot withdrawal in response to mechanical indentation of the plantar surface of each hindpaw using a sharp, cylindrical probe with a uniform tip diameter of approximately 0.2 mm (ALMEMO 2390-5 Electronic von Frey Anesthesiometer; IITC Life Science, San Diego, CA, USA). The probe was applied to six designated loci distributed over the plantar surface of each foot. The minimal force (in grams) that induced paw withdrawal was read off in the display. Two feet were measured in each rat and showed similar mechanical sensitivity in the conditions of control and DNP. The threshold of mechanical withdrawal in each animal was calculated by averaging the 12 (6 × 2) readings, and the force was converted into milli-newtons (mN). The protocols used for determining the pain-related behaviors are similar to those we have previously described [32, 33].

Thermal hyperalgesia was assessed by measuring foot withdrawal latency to heat stimulation. An analgesia meter (IITC Model 336 Analgesia Meter, Series 8; IITC Life Science, San Diego, CA, USA) was used to provide a heat source. In brief, each animal was placed in a box containing a smooth, temperature-controlled glass floor. The heat source was focused on a portion of the hindpaw, which was flushed against the glass, and a radiant thermal stimulus was delivered to that site. The stimulus was shut off when the hindpaw moved (or after 30 s to prevent tissue damage). The intensity of the heat stimulus was maintained throughout all experiments. The elicited paw movement occurred at latency between 10 and 15 s in control animals. Thermal stimuli were delivered 3 times to each hindpaw at 5-6 min intervals. The threshold of thermal hyperalgesia in each animal was calculated by averaging the 6 (3 × 2) readings.

### 2.4. Treatments in Diabetic Neuropathic Pain Model

VBC containing B1, B6, and B12 was first dissolved in physiological saline (pH 7.35) and administered by i.p. injection (B1/B6/B12 = 100/100/2 mg/kg) at doses based on the animal's body weight. VBC was injected daily for 7 consecutive days from day 36 to day 42 after STZ injection. The doses of VBC were used based on our previous studies [19, 20, 27]. Pain behavior tests were done 10–60 min prior to VBC injection on the test days to avoid any acute effect caused by injection.

### 2.5. Histology and Confocal Immunofluorescence Analyses

Deeply anesthetized rats were perfused transcardially with 0.9% saline followed by 4% paraformaldehyde. Bilateral L4 and L5 DRGs were removed and postfixed in 4% paraformaldehyde for 24–48 h. After fixation, the tissues were transferred into 40% sucrose (in 0.1 M PB) for 3 days. The tissues were sectioned at 30 μm thickness. For immunofluorescence staining, free-floating sections were blocked in PBS containing 10% donkey serum with 0.3% Triton X-100 for 2 h and incubated in primary antibody at 4°C overnight. Sections were then washed in 0.1 M PBS with 0.05% Triton X-100, pH 7.6 (3 × 5 min) followed by incubating in the secondary antibody at room temperature for 2 h and washing. Sections were mounted on slides and covered with 90% glycerin for observation under a confocal microscope (FluoView FV1000; Olympus, Shinjuku, Tokyo, Japan). The antibodies used included anti-P2X3 (1 : 1000; Neuromics; Edina, MN, USA), anti-TRPV1 (1 : 200; Santa Cruz, CA, USA), FITC-conjugated Isolectin B4 (1 : 100; Sigma-Aldrich, St. Louis, MO, USA), and anti-CGRP (1 : 100; Abcam, Cambridge, UK). To obtain quantitative measurements of P2X3 and TRPV1 immunofluorescence in the DRG, 20 sections from 4 rats in each group were photographed at the same exposure settings and analyzed. The average red and/or green fluorescence intensity of each pixel was normalized to the background intensity in the same image. Numbers of the CGRP-positive and IB4-positive cells with immunofluorescence stains of P2X3 and TRPV1 were determined by counting ∼1,200 cells per group. Data were analyzed with NIH ImageJ program.

### 2.6. Western Blot

Bilateral L4 and L5 DRGs were quickly removed from deeply anesthetized rats and stored at 80°C. Sequential precipitation procedures were used on the tissue samples that were lysed in ice-cold (4°C) NP-40 lysis buffer containing a mixture of protease inhibitor, phosphatase inhibitors, and phenylmethylsulfonyl fluoride (Sigma-Aldrich). The protein concentrations of the lysates were quantified using the BCA method (with reagents from Pierce), and the total protein content between samples was equalized. Protein samples were separated by SDS-PAGE and transferred to PVDF membrane (both from Bio-Rad Laboratories). The following primary antibodies were used: anti-P2X3 (1 : 1000; Neuromics, Edina, MN, USA), anti-TRPV1 (1 : 100; Santa Cruz), and *β*-actin (1 : 2000; Bioworld, St. Louis Park, MN, USA). The membranes were then developed by enhanced chemiluminescence reagents (PerkinElmer, Waltham, MA, USA) with horseradish peroxidase-conjugated secondary antibodies (R&D Systems, Minneapolis, MN, USA). Data were analyzed with ImageJ.

### 2.7. ELISA

Spinal cord tissue samples at lumbar 4-5 segments were homogenized in ice-cold (4°C) NP-40 lysis buffer containing a mixture of protease inhibitor, phosphatase inhibitors, and phenylmethylsulfonyl fluoride (Sigma-Aldrich). The protein concentrations of the lysates were quantified using the BCA method (Pierce, Dallas, TX, USA), and the total protein content between samples was equalized. Determination of NGF, TNF-*α*, and IL-1*β* levels were measured by enzyme-linked immunosorbent assays (R&D Systems) according to the manufacturer's instructions.

### 2.8. Statistical Analyses

SPSS Rel 15 (SPSS Inc., Chicago, IL, USA) was used to conduct all statistical analyses. Alterations of detected mRNA and protein expression and the behavioral responses to mechanical and thermal stimuli over time among groups were tested with 1-way and 2-way ANOVA with repeated measures followed by Bonferroni's post hoc tests, respectively. Individual Student's *t*-tests were used for Western blot data to test specific hypotheses about differences between each operated or drug-treated group and its corresponding control sham-operated or drug-treated group. All data are presented as the mean ± SEM. The criterion for statistical significance was a *P* value less than 0.05.

## 3. Results

### 3.1. Systematic VBC Administration Attenuated Thermal Hyperalgesia and Mechanical Allodynia in Rats with Hyperglycemia after Injection of STZ

**Figure 1:**
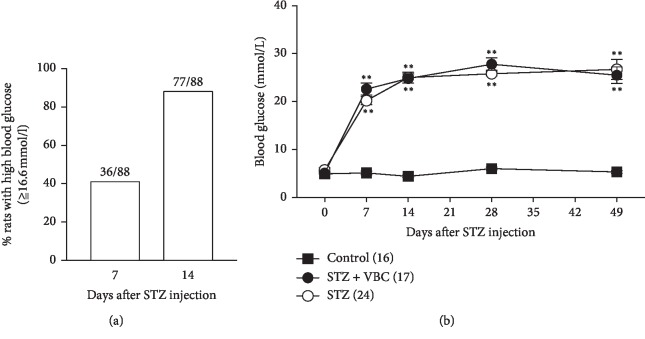
High blood glucose in rats following intraperitoneal injection of streptozotocin (STZ). (a) Percentage of animals (*n* = 88) exhibiting high blood glucose tested on day 7 and 14 after STZ injection. (b) Systematic injection of VBC did not change the STZ-induced increase of glucose. Numbers of animals corresponding to testing day in each group are indicated in the parentheses. Two-way ANOVA, ^*∗∗*^*P* < 0.01 versus control.

**Figure 2:**
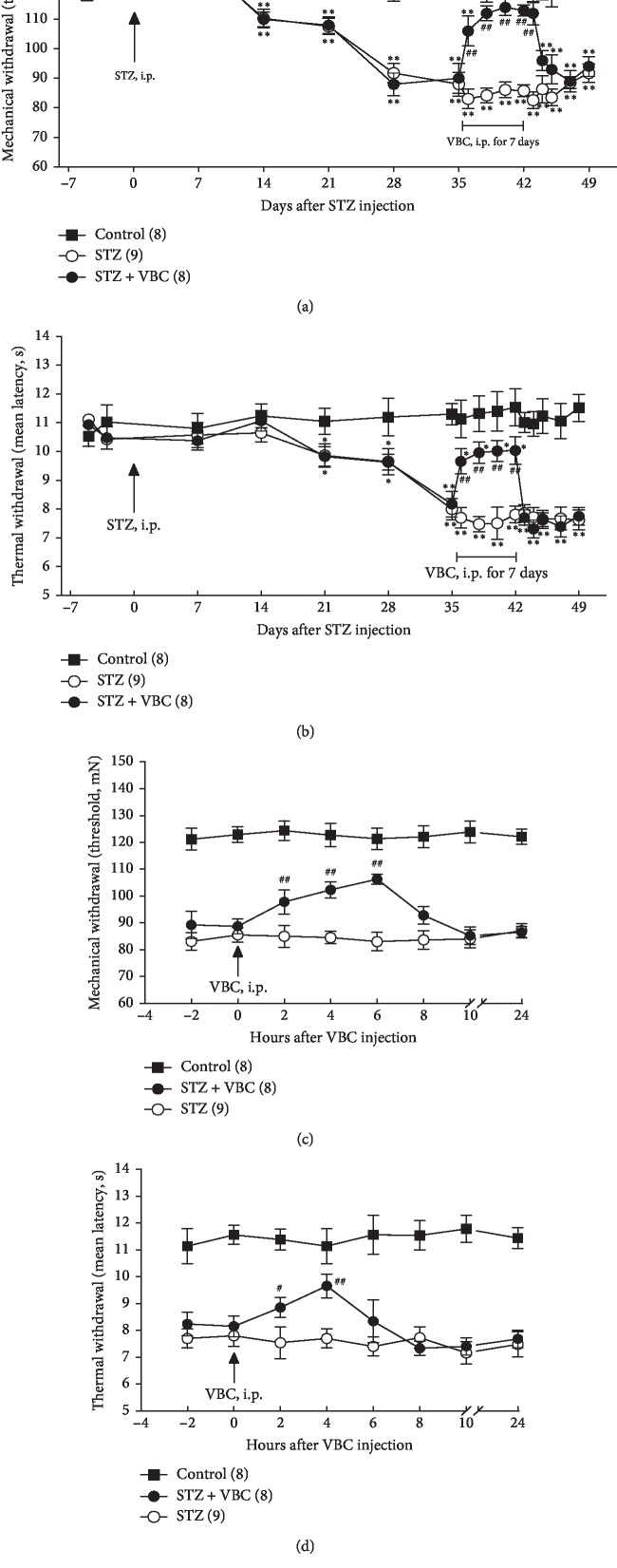
Mechanical allodynia and thermal hyperalgesia after intraperitoneal injection of streptozotocin (STZ) and effects of VBC treatment on the allodynia and hyperalgesia. Development of STZ-induced mechanical hypersensitivity (allodynia) manifested as significant decrease of the mechanical withdrawal thresholds (a) and thermal hyperalgesia manifested as significant shortened latency of thermal withdrawal (b) and long-lasting inhibitory effects of i.p. VBC on the allodynia and thermal hyperalgesia. Immediate inhibitory effects of VBC on STZ-induced mechanical allodynia (c) and thermal hyperalgesia (d). Numbers of animals corresponding to testing day in each group are indicated in the parentheses. Two-way ANOVA, ^*∗∗*^*P* < 0.01 versus control. Student's *t*-test, ^#^*P* < 0.05; ^##^*P* < 0.01 vs. the testing point on day 35 after STZ (a, b) and immediately before the first dose of VBC treatment at point zero (c, d).

Following STZ injection (i.p. 70 mg/kg), 77 out of 88 (87.5%) SD rats developed high blood glucose (>16.6 mmol/L) in 7–14 days. The rest 11 rats (12.5% of 88) did not develop high blood glucose during 2–4 weeks, and these animals were not included in the following studies (Figure 1(a)). The increased blood glucose level in the 77 animals remained high (>16.6 mmol/L) during the entire testing period from 7 or 14 to 49 days after STZ injection, and the glucose levels were not affected by VBC treatment (Figure 1(b)). In addition to the high level of blood glucose, an STZ rat judged with diabetic neuropathic pain had to satisfy another condition, i.e., hypersensitivity to thermal and/or mechanical stimulation, i.e., thermal hyperalgesia and mechanical allodynia. The results showed that 54/77 (70.13%) STZ rats with high glucose were starting to exhibit mechanical allodynia at the testing points of 14^th^ day and/or thermal hyperalgesia at the 21^st^–28^th^ testing points after STZ injection manifested as the significantly decreased mechanical withdrawal threshold and the shortened withdrawal latency (Figures 2(a) and 2(b)). The left 22 rats exhibiting neither thermal hyperalgesia nor mechanical allodynia were not included in the following experiments. Two-way ANOVA followed by *post hoc* pairwise comparisons was used for statistical analysis. Repeated administration of VBC (B1/B6/B12 = 100/100/2 mg/kg, i.p.), daily for 7 consecutive days during 36 to 42 days after STZ injection, significantly attenuated the thermal hyperalgesia and mechanical allodynia in the STZ animals. These inhibitory effects were seen during the period of VBC treatment, and any tolerance was not observed. However, the analgesic effects quickly disappeared one day after termination of VBC treatment (Figures 2(a) and 2(b)). The analgesic effect produced by a single dose of VBC lasted for about 8 h for mechanical allodynia and 6 h for thermal hyperalgesia (Figures 2(c) and 2(d)). These results support the idea that systematic administration of VBC can attenuate pain in STZ-DNP rats and further confirm the results we have demonstrated in a recent study [27].

### 3.2. Systematic VBC Administration Suppressed the Increased Expression of P2X3 and TRPV1 in the Diabetic DRGs

**Figure 3:**
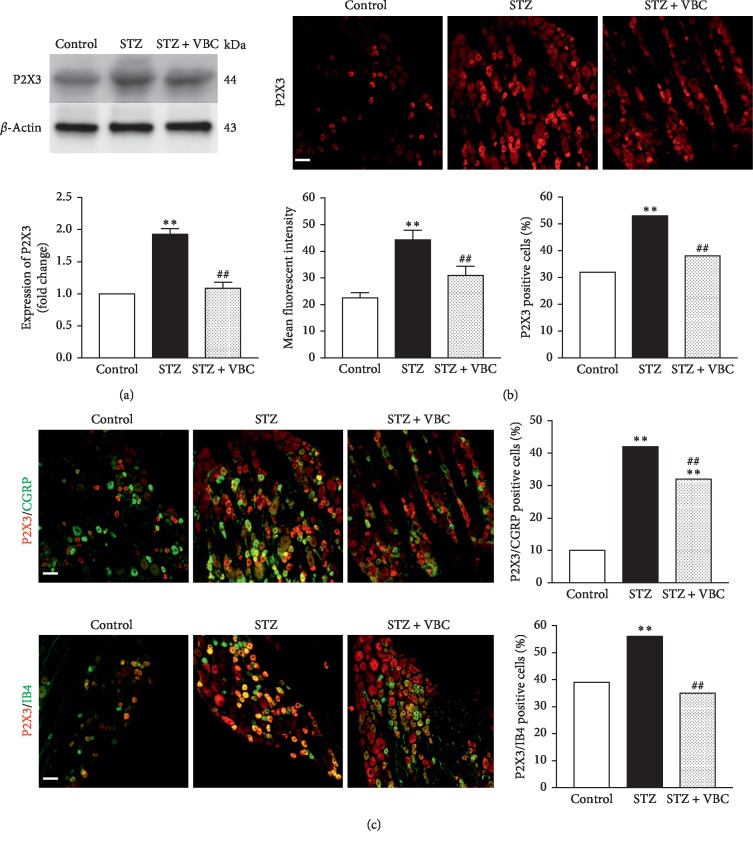
Protein expression and immunofluorescent staining of P2X3 in DRG neurons in painful STZ rats and effects of VBC treatment on P2X3 expression. (a) Western blot analysis showing expression of P2X3 in DRGs. Top: representative bands; bottom: data summary. (b) Immunofluorescent staining showing expression of P2X3. Top: representative staining; bottom: data summary showing the man fluorescent intensity and P2X3-positive cells, respectively. (c) Expression and colocalization of P2X3 with the CGRP- and IB4-positive neurons. Left: representative immunofluorescent staining; right: data summary. (a–c) Tissues were taken on day 28 after vehicle control, STZ (group STZ) or 4-5 h after VBC treatment (group STZ + VBC). Four DRG samples (each with two DRGs at L4 and L5 from the same rats) from 4 rats were included in each of the groups (a). To obtain quantitative measurements of P2X3 immunofluorescence in the DRG, 20 sections from 4 rats (8 DRGs) in each group were photographed at the same exposure settings and analyzed. Numbers of the CGRP- and IB4-positive cells with immunofluorescence stains of P2X3 were determined by counting 1,000–1,200 cells per group. One-way ANOVA, ^*∗∗*^*P* < 0.01 versus control group; ^##^*P* < 0.01 versus STZ group.

**Figure 4:**
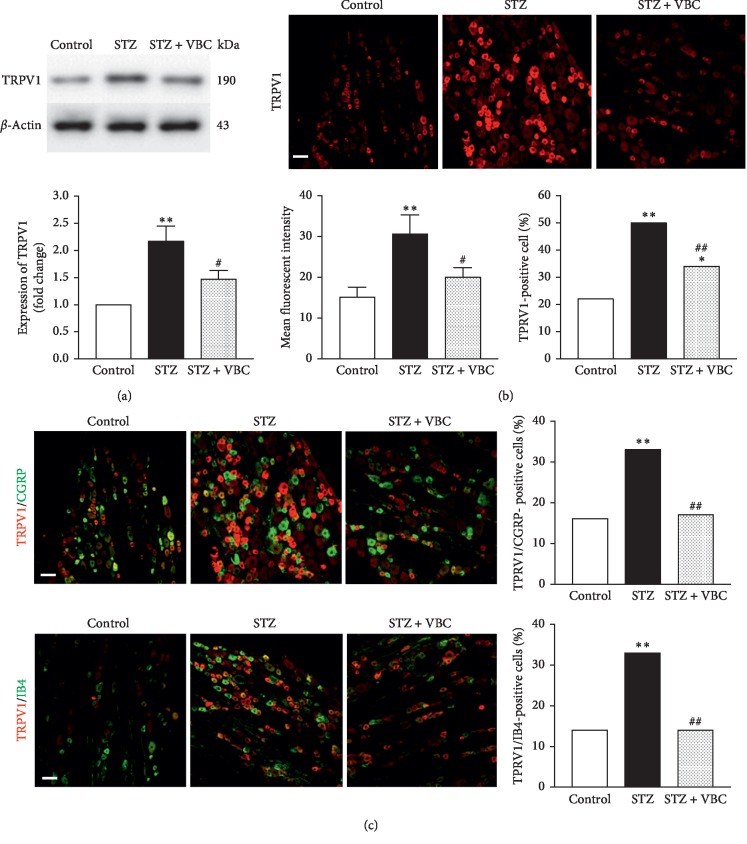
Protein expression and immunofluorescent staining of TRPV1 in DRG neurons in painful STZ rats and effects of VBC treatment on TRPV1 expression. (a) Western blot analysis showing expression of TRPV1 in DRGs. Top: representative bands; bottom: data summary. (b) Immunofluorescent staining showing expression of TRPV1. Top: representative staining. Bottom: data summary showing the man fluorescent intensity and TRPV1-positive cells, respectively. (c) Expression and colocalization of TRPV1 with the CGRP- and IB4-positive neurons. Left: representative immunofluorescent staining; right: data summary. (a–c) Tissues were taken on day 28 after vehicle Control, STZ (group STZ) or 4-5 h after VBC treatment (group STZ + VBC). Four DRG samples (each with two DRGs at L4 and L5 from the same rats) from 4 different rats were included in each of the groups (a). To obtain quantitative measurements of TRPV1 immunofluorescence in the DRG, 20 sections from 4 rats (8 DRGs) in each group were photographed at the same exposure settings and analyzed. Numbers of the CGRP- and IB4-positive cells with immunofluorescence stains of TRPV1 were determined by counting 1,000–1,200 cells per group. One-way ANOVA, ^*∗∗*^*P* < 0.01 versus control group; ^#^*P* < 0.05; ^##^*P* < 0.01 versus STZ group.

In addition to inducing behaviorally expressed neuropathic thermal hyperalgesia and mechanical allodynia, STZ-induced hyperglycemia and DNP caused significantly increased expression of P2X3 and TRPV1 in the DRG. Western blotting and immunohistochemistry analyses showed that expression of P2X3 protein was significantly increased in STZ-DRG with high glucose and DNP (Figures 3(a) and 3(b)). The increased P2X3 protein immunoreactivity was distributed in both the IB4- and CGRP-positive, small DRG neurons (Figure 3(c)). Repeated administration of VBC (B1/B6/B12 = 100/100/2 mg/kg, i.p.) for 7 consecutive days significantly inhibited STZ-induced expression of P2X3 (Figures 3(a)–3(c)). Similarly, TRPV1 protein was also significantly increased in STZ-DRG with high glucose and DNP (Figures 4(a) and 4(b)). The increased TRPV1 immunoreactivity was distributed in both IB4- and CGRP-positive, small DRG neurons (Figure 4(c)). However, the rate of expression of P2X3 and TRPV1 are different. The increased expression of TRPV1 induced by STZ injection in CGRP-positive neurons was significantly lower than that of P2X3 (TRPV1 from ∼17% to 33% vs P2X3 from 10% to 42%), while in IB4-positive neurons, the increased expression of TRPV1 was significantly higher than that of P2X3 (TRPV1 from approximately 14% to 33% vs. P2X3 from 39% to 56%). Repeated administration of VBC (B1/B6/B12 = 100/100/2 mg/kg, i.p.) for 7 consecutive days also significantly inhibited STZ-induced expression of TRPV1 (Figures 4(a)–4(c)). These results indicate that VBC treatment can inhibit the key molecules P2X3 and TRPV1 that play critical roles in painful conditions, in addition to the behavioral painful syndromes in STZ-DNP rats.

### 3.3. Systematic VBC Administration Reduced Levels of NGF, TNF-*α*, and IL-1*β* in the Diabetic Spinal Cords

**Figure 5:**
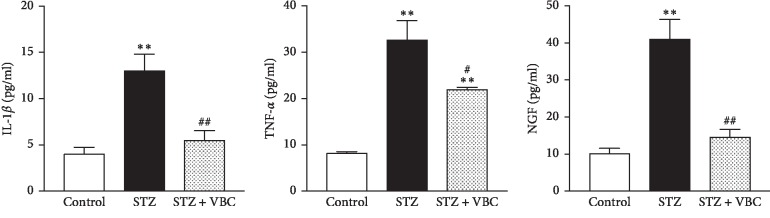
Systematic administration of VBC inhibits STZ-induced activity of IL-1*β*, TNF-*α*, and NGF in the spinal cord in painful STZ rats. Tissues were collected 4–6 hours after the VBC treatment. Tissues were taken on day 28 after vehicle control, STZ, or 4-5 h after VBC treatment (group STZ + VBC). Four samples (each with one spinal segment at L4-L5) from 4 rats were included in each of the groups. One-way ANOVA, ^*∗∗*^*P* < 0.01 versus control. ^#^*P* < 0.05; ^##^*P* < 0.01 versus STZ.

Inflammatory cytokines interleukine-1*β* (IL-1*β*), tumor necrosis factor-*α* (TNF-*α*), and nerve growth factor (NGF) were reported to be associated with DNP in previous studies [18, 34, 35]. By means of ELISA, we measured IL-1*β*, TNF-*α*, and NGF from the L4-L5 spinal cord tissues. The levels of IL-1*β* (STZ 12.99 ± 1.83 pg/ml vs. sham 4.00 ± 0.74 pg/ml), TNF-*α* (STZ 32.61 ± 4.26 pg/ml vs. sham 8.13 ± 0.41 pg/ml), and NGF (STZ 41.02 ± 5.34 pg/ml vs sham 10.07 ± 1.48 pg/ml) were significantly increased in the spinal cord in painful STZ animals. Repeated administration of VBC (B1/B6/B12 = 100/100/2 mg/kg, i.p.) for 7 consecutive days from day 36 to day 42 after STZ injection significantly reduced STZ-induced increase of IL-1*β*, TNF-*α*, and NGF. Data are summarized in Figure 5.

## 4. Discussion

In this study, we investigated the analgesic effect of the combination of B1/B6/B12 (VBC) and its effects on the expression of P2X3 and TRPV1 receptors in DRG neurons and the inflammatory cytokines IL-1*β*, TNF-*α*, and NGF in the spinal cord in STZ-induced diabetic rats. Our results have demonstrated that systemic administration of VBC can significantly attenuate thermal hyperalgesia and mechanical allodynia as well as inhibiting the increased expression of P2X3 and TRPV1 in DRG neurons and the level of inflammatory cytokines IL-1*β*, TNF-*α*, and NGF in the spinal cord in STZ-induced diabetic rats. These findings support a strategy of managing chronic DNP by long-term VBC treatment and suggest that P2X3 and TRPV1 in the DRG, and IL-1*β*, TNF-*α*, and NGF in the lumbar spinal cord may play important roles in the pathogenesis of DNP.

It has been considered that hyperglycemia results in damage of peripheral nerve terminals, leading to diabetes mellitus patients complain of pain [36–38], although the specific cellular and molecular mechanisms that underlie the pathogenesis of DNP remain elusive. However, in this study, our results show that systemic treatment of VBC suppresses STZ-induced thermal hyperalgesia and mechanical allodynia without affecting the blood glucose level, which was previously quickly increased following injection of STZ. These findings indicate that VBC's effect in attenuating diabetic pain is independent of blood glucose level and suggest that hyperglycemia itself may not be sufficient to maintaining DNP. In addition, this study also showed that ∼30% (22/77) rats with hyperglycemia did not show any painful syndromes, suggesting that hyperglycemia itself is also not sufficient to inducing DNP. Our ongoing experiments are investigating the possible mechanisms underlying the roles of hyperglycemia in the production and persistence of DNP. Our results show that a single dose of VBC can produce significant analgesic effect, which starting within 2 h and lasting for 6–8 h. Repetitive treatment for a week can produce consistent analgesia during the period of VBC administration. However, such beneficial effects of VBC treatment quickly disappeared within one day of VBC termination, suggesting that such long-term VBC treatment does not produce accumulative effect and long-term treatment is necessary for the management of chronic DNP. The good thing is that repetitive, long-term treatment (up to 15 years) of B vitamins, which are water soluble, does not produce any detectable side effects in human subjects [39].

P2X3 receptor and transient receptor potential vanilloid 1 (TRPV1) ion channel are highly expressed in DRG neurons and play important roles in pain signal transduction. Activation of both P2X3 and TRPV1 has been linked to chronic inflammatory pain conditions and peripheral neuropathy, as observed in diabetes-induced neuropathy. Activation of P2X3 receptor mediates primarily the mechanical sensation, while TRPV1 serves as a multimodal sensor of noxious stimuli such as heat, protons, capsaicin, and a variety of endogenous lipids termed endovanilloids [40–46]. Our results show that expression of P2X3 and TRPV1 is significantly increased in STZ rats with neuropathic painful conditions exhibited as thermal hyperalgesia and mechanical allodynia. Such increased expression of P2X3 and TRPV1 can be significantly reduced by repetitive systematic VBC treatment.

The neurotrophin nerve growth factor (NGF) plays a critical role in the regulation of both innate and acquired immunity. NGF is released in the process of inflammation which leads to increased pain perception. TRPV1 and substance P are known to be regulated by NGF and have been related to diabetes-induced neuropathy [34, 35, 47, 48] Mediators of inflammation, particularly the proinflammatory cytokines IL-1*β* and TNF-*α* play critical roles in various neuropathic pain conditions [49–52]. These inflammatory molecules are released from the main immune cells in the CNS, microglia, and astrocytes, when they become activated [53, 54]. Our results show that IL-1*β*, TNF-*α*, and NGF were significantly increased in the lumbar spinal cord of STZ-induced rats with diabetic pain. VBC treatment greatly reduces the levels of IL-1*β*, TNF-*α*, and NGF in the spinal cord, in addition to suppressing the thermal hyperalgesia and mechanical allodynia. These results demonstrate that VBC has analgesic effects in DNP treatment and such analgesia may be mediated by inhibition of VBC on the pain-related mediators and inflammatory cytokines including P2X3, TRPV1, TNF-*α*, IL-1*β*, and NGF. These findings also suggest that P2X3 and TRPV1 in the DRG as well as TNF-*α*, IL-1*β*, and NGF in the spinal cord may play important roles in the pathogenesis of DNP, and that these molecules may serve as potential therapeutic targets for managing diabetic neuropathic pain.

## Data Availability

All data used to support the findings of this study are included within the article.
